# Phytochemical analysis, antioxidant, antimicrobial, and anti-enzymatic properties of *Alpinia coriandriodora* (sweet ginger) rhizome

**DOI:** 10.3389/fpls.2023.1284931

**Published:** 2023-10-23

**Authors:** Xia Wu, Feng Wei, Furong Ding, Nian Yang, Jingming Niu, Yuanquan Ran, Minyi Tian

**Affiliations:** ^1^ Key Laboratory of Plant Resource Conservation and Germplasm Innovation in Mountainous Region (Ministry of Education), School of Liquor and Food Engineering, Guizhou University, Guiyang, China; ^2^ National & Local Joint Engineering Research Center for the Exploitation of Homology Resources of Southwest Medicine and Food, Guizhou University, Guiyang, China; ^3^ First Affiliated Hospital of Guizhou University of Traditional Chinese Medicine, Guiyang, China

**Keywords:** *Alpinia coriandriodora*, phytochemical analysis, antioxidant, antimicrobial, enzyme inhibition

## Abstract

*Alpinia coriandriodora*, also known as sweet ginger, is a medicinal and edible plant. *A. coriandriodora* rhizome is popularly utilized in traditional Chinese medicine and as flavouring spices, but there are few reports on its constituents and bioactivities. This study analyzed the phytochemical components of *A. coriandriodora* rhizome by GC-MS and UHPLC-Q-Orbitrap-MS and evaluated its antioxidant, antimicrobial, and anti-enzymatic properties. According to the GC-FID/MS data, its rhizome essential oil (EO) consisted mainly of (*E*)-2-decenal (53.8%), (*E*)-2-decenyl acetate (24.4%), (*Z*)-3-dodecenyl acetate (3.5%), and (*E*)-2-octenal (3.5%). Its water extract (WE) and 70% ethanol extract (EE) showed high total phenolic content (TPC, 52.99–60.49 mg GAEs/g extract) and total flavonoid content (TFC, 260.69–286.42 mg REs/g extract). In addition, the phytochemicals of WE and EE were further characterized using UHPLC-Q-Orbitrap-MS, and a total of sixty-three compounds were identified, including fourteen phenolic components and twenty-three flavonoid compounds. In the antioxidant assay, WE and EE revealed a potent scavenging effect on DPPH (IC_50_: 6.59 ± 0.88 mg/mL and 17.70 ± 1.15 mg/mL, respectively), surpassing the BHT (IC_50_: 21.83 ± 0.89 mg/mL). For the antimicrobial activities, EO displayed excellent antibacterial capabilities against *Proteus vulgaris*, *Enterococcus faecalis*, *Bacillus subtilis*, *Escherichia coli*, and *Staphylococcus aureus* with DIZ (12.60–22.17 mm), MIC (0.78–1.56 mg/mL), and MBC (3.13 mg/mL) and significantly inhibited *Aspergillus flavus* growth (MIC = 0.313 mg/mL, MFC = 0.625 mg/mL, respectively). In addition to weak tyrosinase and cholinesterase inhibition, EE and WE had a prominent inhibitory effect against α-glucosidase (IC_50_: 0.013 ± 0.001 mg/mL and 0.017 ± 0.002 mg/mL), which was significantly higher than acarbose (IC_50_: 0.22 ± 0.01 mg/mL). Hence, the rhizome of *A. coriandriodora* has excellent potential for utilization in the pharmaceutical and food fields as a source of bioactive substances.

## 1 Introduction

*Alpinia* (Zingiberaceae) has approximately 250 species and is mostly distributed in subtropical and tropical Asia (WFO, 2023). *Alpinia* is widely cultivated for its rhizome, which is consumed in fields of folk medicine, condiment, perfume, and food (Yang and Eilerman, 1999; Victório, 2011; Ibrahim et al., 2014). *Alpinia* plants are abundant in essential oil (EO) and are frequently used to treat cancer, ulcer, neural disorders, diabetes, and Alzheimer’s disease (Ghosh and Rangan, 2013; Rajasekar et al., 2014; Shi et al., 2015). Numerous bioactivities of the *Alpinia* plant’s essential oils and extract have been reported, including anti-inflammatory, antimicrobial, insecticidal, neuroprotective, antioxidant, antifungal, and analgesic activities (Ghosh and Rangan, 2013; Van et al., 2021).

*Alpinia coriandriodora* D. Fang., also known as sweet ginger, is a perennial plant with a distinctive coriander fragrance that is extensively cultivated in China due to its medicinal and edible value (Wu and Larsen, 2000; Cheng et al., 2021). *A. coriandriodora* rhizome is used as traditional Chinese medicine for treating fever, asthma, cold, stomachache, and indigestion (CHMC-Chinese Herbal Medicine Company, 1994; Zhang and Li, 2015; Wu et al., 2016). Moreover, its rhizome was utilized as spices in the preparation of soups and stews. Interestingly, wrapping zongzi with its leaves is a specialty of residents in Guangxi, China. In previous studies on *A. coriandriodora* rhizome, several diarylheptanoids and flavonoids were isolated, and these compounds had intracellular antioxidant and anti-inflammatory actions by inhibiting NO release (Cheng et al., 2021). *A. coriandriodora* is rich in essential oil, and its EO has been used in traditional medicine (Tran et al., 2022). Previous research indicated that *A. coriandriodora* rhizome EO was mainly composed of (*E*)-2-decenal (60.4–56.3%) and (*E*)-2-decenyl acetate (19.6–4.3%) and possessed potent anticancer and anti-*malassezia* effects (Hong et al., 2022; Tran et al., 2022).

Despite its high edible and medicinal value, there are few reports on the phytochemistry and biological activities of *A. coriandriodora* rhizome, which may hinder its utilization. Thus, this study analyzed the phytochemical constitution of *A. coriandriodora* rhizome using GC-FID/MS and UHPLC-Q-Orbitrap-MS and evaluated its antioxidant, antimicrobial, and anti-enzymatic properties.

## 2 Materials and methods

### 2.1 Chemical and reagents

Streptomycin, Folin-Ciocalteu reagent, resazurin, galanthamine, kojic acid, acarbose, rutin, and gallic acid were from Solarbio Life Sciences (Beijing, China). Sigma-Aldrich (Germany) provided BTCl (butyrylthiocholine chloride), ATCI (acetylthiocholine iodide), L-tyrosine, *p*-NPG (*p*-nitrophenyl-α-D-glucopyranoside), BChE (butyrylcholinesterase), AChE (acetylcholinesterase), tyrosinase, α-glucosidase, ABTS (2,2-azino-bis-3-ethylbenzthiazoline-6-sulphonic acid), DPPH (1,1-diphenyl-2-picrylhydrazyl), BHT (butylated hydroxytoluene), and ascorbic acid. Merck (Darmstadt, Germany) supplied a standard n-alkane (C_7_–C_30_) in the GC-MS test and formic acid and acetonitrile (LC/MS-grade) in the UHPLC-Q-Orbitrap-MS test. Aladdin (Shanghai, China) offered other solvents and chemicals in analytical purity.

### 2.2 Plant material

*Alpinia coriandriodora* D. Fang was cultivated at Guigang City, Guangxi Province, China (latitude: 23°80′89.74′′N and longitude: 110°18′64.68″E). The plant samples were harvested in August 2020 and authenticated by Professor Guoxiong Hu, a plant taxonomist. A voucher specimen has been deposited at the Herbarium of the College of Life Sciences, Guizhou University, China (Voucher No: AC2020803).

### 2.3 Extraction of EO, WE, and EE

Hydro-distillation was employed to prepare the EO of *A. coriandriodora* rhizome. In brief, the fresh rhizome (2 kg) was washed using distilled water, crushed, and hydrodistilled (4 h) in a Clevenger’s apparatus. After drying with anhydrous Na_2_SO_4_, EO was stored at 4°C until further use.

Reflux extraction was used to produce the WE and EE of the *A. coriandriodora* rhizome. The crushed plant material (0.5 kg) and 70% ethanol or distilled water (2 L) were added to a round-bottomed flask (5 L). Afterward, the mixture was reflux-extracted twice (2 h/reflux). The extraction solution was mixed, filtered, condensed using a rotary evaporator, and dried using a freeze drier. Then, WE and EE were weighed and stored in a brown glass bottle at 4°C until usage.

### 2.4 EO’s composition analysis using GC-FID/MS

Quantitative analysis of phytochemical components was performed on GC-FID (Agilent Technologies, 6890N) equipped with an HP-5MS column (60 m × 0.25 mm, 0.25 mm film thickness). The helium was used as a carrier gas (1 mL/min). The injection volumes were 1 µL (split ratio 1:20). The injector temperature was 250°C. The oven temperature was programmed as follows: kept at 70°C (2 min), raised to 180°C (2°C/min), increased to 310°C (10°C/min), and held at 310°C for 14 min (running time: 84 min). Agilent 6890/5975C GC-MS was employed for qualitative analysis, and its GC settings were the same as those of GC-FID. The following conditions were applied in MS: ion source (EI, 70 eV); ion source temperature (230°C); interface temperature (280°C); scan range from 29 to 500 *m/z.* The peak area revealed the relative percentage of different chemical constituents. A series of n-alkanes (C_8_–C_16_) were used to determine the retention index (RI). Matching the RI and MS information in Wiley 275 and NIST 2020 databases was done to identify the chemical constituents.

### 2.5 Determination of total phenolic and flavonoid contents

#### 2.5.1 TPC analysis

The TPC in WE and EE were quantified using the Folin-Ciocalteu method (Tian et al., 2020). Folin-Ciocalteu reagent (2.5 mL) and sample solution (0.5 mL) were combined and kept at 25°C for 5 min. The 7.5% Na_2_CO_3_ solution (4 mL) was subsequently incorporated and reacted for 1 h at 25°C. The absorbance at 760 nm was measured, and gallic acid equivalents (mg GAEs/g extract) were used to express TPC.

#### 2.5.2 TFC analysis

The TFC in WE and EE were quantified using NaNO_2_-Al(NO_3_)_3_-NaOH colorimetry with minor modifications (China Pharmacopoeia Committee, 2020). The sample solution (5 mL) and NaNO_2_ (0.4 mL, 5%) were blended and kept at 25°C for 5 min, which was reacted with 10% Al(NO_3_)_3_ solution (0.4 mL) for 5 min. Subsequently, 4 mL NaOH solution (4%) and distilled water were added to gain a final volume of 10 mL and reacted 15 min at 25°C. The absorbance at 510 nm was measured, and rutin equivalents (mg REs/g extract) were applied to represent TFC.

### 2.6 Identification of phytochemicals in WE and EE by UHPLC-Q-Orbitrap-MS

UHPLC-Q-Orbitrap-MS (ultra-high-performance liquid chromatography coupled with quadrupole orbitrap mass spectrometer) was employed for the phytochemical analysis of WE and EE. Dionex Ultimate 3000 UHPLC system settings were as follows: Hypersil Gold C_18_ column (2.1 mm × 100 mm, 1.9 μm), injection volume (5 μL), column temperature (40°C), flow rate (0.3 mL/min), and mobile phases (A: 0.1% formic acid water solution and B: 0.1% formic acid acetonitrile). The gradient elution protocol was as follows: 5% B (0–2 min), 5–95% B (2–42 min), 95% B (42–47 min), 5% B (47.1 min), and 5% B (47.1–50 min).

The MS data collection was performed using Q-Orbitrap-MS (Q Exactive Focus hybrid quadrupole-Orbitrap high-resolution mass spectrometry, Thermo Fisher Scientific) with HESI-II (heated electrospray ionization). The HESI-II settings were as follows: spray voltages –2.5/+ 3.0 kV, sheath gas 35 arb, auxiliary gas 10 arb, sweep gas 0 arb, RF lens amplitude 60, vaporizer temperature 350°C, and capillary temperature 320°C. The mode of full mass/ddMS2 mode was used, and its parameters were as follows: full scan range (*m/z* 100 to 1,500); resolution MS1 (70,000) and MS2 (17,500); maximum injection time 100 ms (MS1) and 50 ms (MS2); stepped normalized collision energy (20/40/60 eV); automatic gain control (AGC) target values 1e^6^ (MS1) and 2e^5^ (MS2). Thermo Fisher Scientific’s Xcalibur 4.1 was used to process the mass spectrum data of the chemical composition. The identification of phytochemical constituents was determined by comparing mzVault and mzCloud databases and references within a 10 ppm threshold.

### 2.7 Antioxidant properties

The radical scavenging capacities on DPPH (1,1-diphenyl-2-picrylhydrazyl) and ABTS (2,2-azino-bis-3-ethylbenzthiazoline-6-sulphonic acid) were assessed according to the method described in the literature (Tian et al., 2020). Butylated hydroxytoluene (BHT) was used as a positive control.

#### 2.7.1 DPPH assay

Equal amounts of DPPH solution (0.08 mM) and sample solution (100 μL) were mixed, and the reaction was carried out at 25°C for 30 min away from light. Optical density at 517 nm was measured, and IC_50_ values and BHT equivalents (mg BEs/g sample) were calculated for demonstrating the scavenging effect of DPPH.

#### 2.7.2 ABTS assay

In order to produce the ABTS•^+^ solution, 50 mL of ABTS solution (0.7 mM) was reacted with an equal amount of K_2_S_2_O_8_ solution (2.45 mM) for 12 h at 25°C without light. In addition, it was further diluted with ethanol prior to use to yield an absorbance of 0.70 ± 0.02 at 734 nm. Then, 0.4 mL of sample solution reacted with 4 mL of diluted ABTS•^+^ solution for 10 min at 25°C away from light. Optical density at 734 nm was gained, and IC_50_ values and the equivalents of BHT (mg BEs/g sample) were expressed in the results.

### 2.8 Antimicrobial properties

#### 2.8.1 Bacterial strains

The antibacterial properties of EO, WE, and EE was assessed against Gram-negative bacterias (*Escherichia coli* CICC 10389, *Enterococcus faecalis* ATCC 19433, and *Proteus vulgaris* ACCC 11002) and Gram-positive bacterias (*Pseudomonas aeruginosa* ATCC 9027, *Bacillus subtilis* ATCC 6633, and *Staphylococcus aureus* ATCC 6538P).

#### 2.8.2 Agar well diffusion assay

The agar well diffusion method assayed the diameter of the inhibition zone (DIZ) (Zhang et al., 2017). EO was dissolved in DMSO and diluted with sterile distilled water (DMSO content was less than 0.5%). Sterile distilled water was used to dissolve the WE and EE. Three sample solutions were brought to a concentration of 100 mg/mL. Streptomycin was dissolved in distilled water to reach a 100 μg/mL concentration, which was used as a positive control. The Mueller-Hinton agar medium surface was spread with 100 μL of the bacterial suspension (1 × 10^7^ CFU/mL) and was covered with filter paper discs of 6 mm diameter (containing 20 μL sample solutions). The DIZ was determined after 24 h of incubation at 37°C.

#### 2.8.3 Determination of MIC and MBC

In a 96-well plate, 100 μL of bacterial suspensions (1 × 10^6^ CFU/mL) was added to an equal amount of sample solution. After 24 h of incubation at 37°C, each well was supplied with 20 μL of resazurin aqueous solution (100 μg/mL), which was cultured for 2 h at 37°C without light. The minimal sample concentration without color change was determined as MIC (minimal inhibitory concentration) (Tian et al., 2020). The mixture (10 μL) from the wells that did not change color was subcultured in a Mueller Hinton agar plate for 24 h at 37°C, the least sample concentration at which no bacterial growth was determined as MBC (minimal bactericidal concentration) (Eloff, 1998).

#### 2.8.4 Antifungal capacity

The anti-*Aspergillus flavus* activity of EO was tested by a previously published method with slight modification (da Silva et al., 2012) using amphotericin B as a positive control. The EO solution (3.125 mg/mL–100 mg/mL) was mixed with Potato Dextrose Agar (PDA) medium (2:18, v:v). Subsequently, the mixture (15 mL) was added to Petri dishes (diameter 90 mm) and solidified. The *A. flavus* spore suspension (1 × 10^3^ CFU/mL, 100 μL) was inoculated into wells (6 mm in diameter) of the culture medium and incubated at 28°C for 36 h. The MIC value was recorded as EO concentrations that inhibited the visible growth of *A. flavus*, and the diameter of colony growth zone was less than 12 mm (including well diameter of 6 mm) (Yadav et al., 2005). The minimum fungicidal concentration (MFC) was the minimum sample concentration showing no *A. flavus* growth on the PDA medium.

### 2.9 Enzyme inhibitory properties

The enzyme inhibitory properties of *A. coriandriodora* rhizome WE, EE, and EO on cholinesterases (AChE and BChE), tyrosinase, and α-glucosidase were tested, and galanthamine, arbutin, and acarbose were used as positive controls, respectively (Tian et al., 2020).

#### 2.9.1 Cholinesterases inhibition

In a 96-well plate, 10 μL of AChE or BuChE solution (pH 8.0, 0.5 U/mL) and 50 μL of sample solution were mixed and incubated for 5 min at 4°C. Subsequently, each well was injected with 20 μL of ATCI (acetylthiocholine iodide) or BTCl (butyrylthiocholine chloride) solution (2 mM) and 20 μL of color-developing agent 5,5’-dithiobis-(2-nitrobenzoic acid) solution (2 mM). After 30 min of reaction at 37°C, the absorbance at 405 nm was read. The galanthamine equivalents (mg GALAEs/g sample) and IC_50_ values expressed cholinesterase inhibition.

#### 2.9.2 Tyrosinase inhibition

In a 96-well plate, 100 μL of tyrosinase solution (100 U/mL) was mixed with 70 μL of sample solution and incubated for 5 min at 37°C. Then, 80 μL of L-tyrosine solution (5.5 mM) was injected into each well. After 30 min of reaction at 37°C, the absorbance at 492 nm was measured. Data were presented as arbutin equivalents (mg AREs/g sample) and IC_50_ values.

#### 2.9.3 α-Glucosidase inhibition

In a 96-well plate, 10 μL of α-glucosidase solution was reacted with 90 μL sample solution for 15 min at 37°C. Next, 10 μL of *p*-NPG (*p*-Nitrophenyl-α-D-glucopyranoside) solution (1 mM) was injected into each well and maintained at 37°C or 15 min. Lastly, 80 μL of Na_2_CO_3_ (0.2 M) was added for stopping the reaction. After the absorbance detection at 405 nm, α-glucosidase inhibition was represented as acarbose equivalents (mmoL ACEs/g sample) and IC_50_ values.

### 2.10 Statistical analysis

All data were collected from three independently performed experiments and were expressed as mean ± standard deviation (SD). In SPSS (version 25) software, the two-tailed unpaired t-test or one-way analysis of variance (ANOVA) and Fischer’s LSD *post hoc* test (*p* < 0.05) were utilized to compare the significant difference between the two groups.

## 3 Results and discussion

### 3.1 Chemical composition of EO

**Figure 1:**
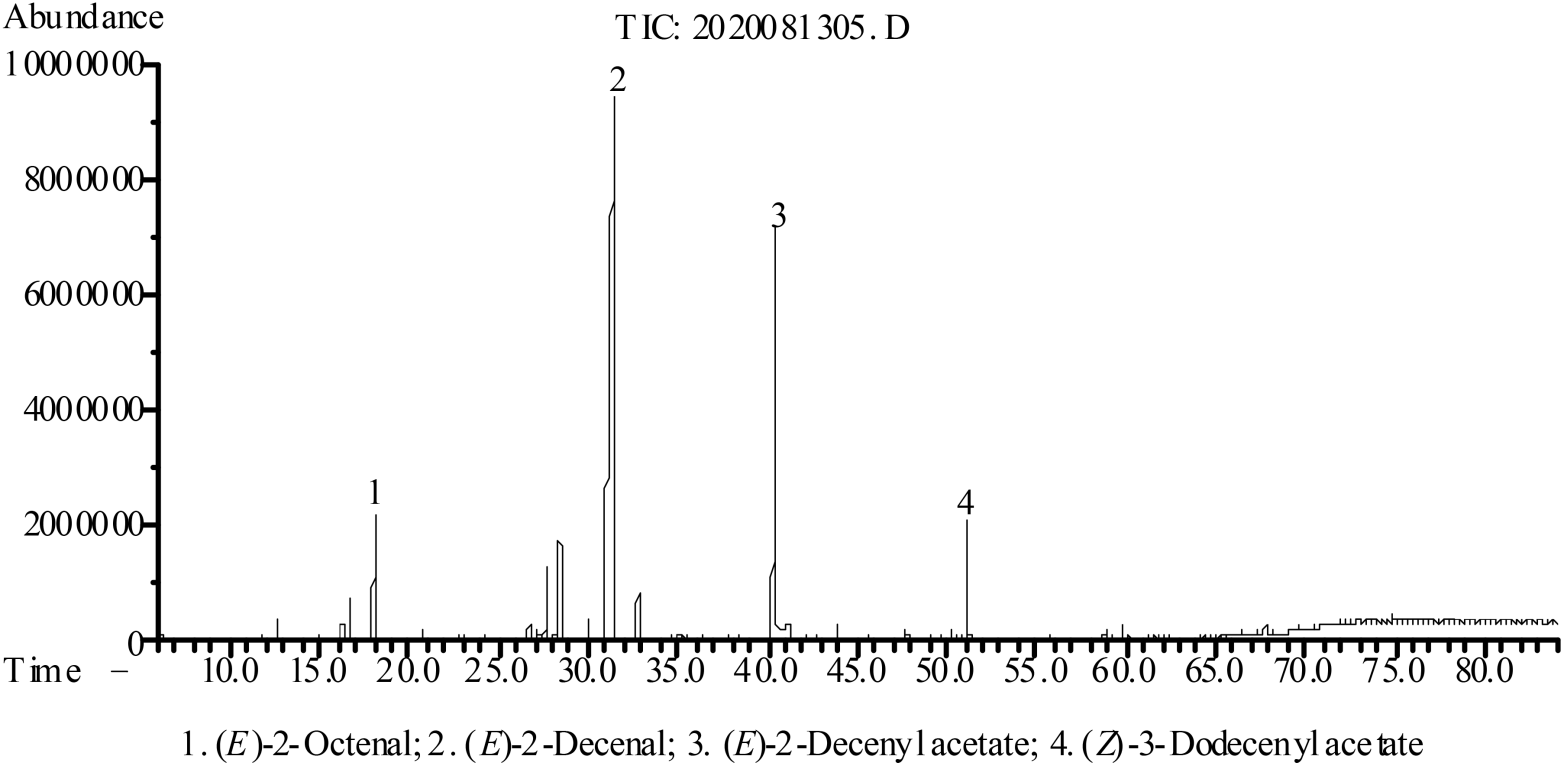
GC-MS chromatogram of *A. coriandriodora* EO.

Based on the fresh weight of *A. coriandriodora* rhizome, the extraction rate of EO was 0.56% (w/w). As shown in **Table 1**, a total of 27 chemical compositions, representing 97.0% of the total EO, were identified by GC-MS/FID. As shown in **Figure 1**, it was mainly composed of (*E*)-2-decenal (53.8%), (*E*)-2-decenyl acetate (24.4%), (*Z*)-3-dodecenyl acetate (3.5%), and (*E*)-2-octenal (3.5%). According to our previous study, EO’s main compounds from *A. coriandriodora* rhizome harvested at the same location (Guangxi, China) in July 2019 were (*E*)-2-decenal (56.3%), (*E*)-2-decenyl acetate (19.6%), (*Z*)-3-dodecenyl acetate (3.8%), and (*E*)-2-octenal (3.6%) (Hong et al., 2022). The main chemical components of *A.coriandriodora* essential oils from July and August are the same, and the difference in their content may be caused by different harvesting times.

**Table 1:** Chemical components of the EO from *A. coriandriodora* rhizome.

Compounds	RT	RI ^a^	RI ^b^	Area (%)	Identification ^c^
Heptanal	10.30	902	901	tr ^d^	MS, RI
*α*-Pinene	11.87	937	937	0.1	MS, RI
Camphene	12.59	953	952	0.4	MS, RI
*δ*-phellandrene	13.91	982	995	tr ^d^	MS, RI
Octanal	14.91	1003	1003	0.2	MS, RI
*p*-Cymene	16.27	1027	1025	0.4	MS, RI
*D*-Limonene	16.52	1031	1031	tr ^d^	MS, RI
Eucalyptol	16.70	1035	1032	1.0	MS, RI
(*E*)-2-Octenal	18.06	1058	1060	3.5	MS, RI
*α*-Pinene oxide	20.71	1104	1095	0.3	MS, RI
*cis*-Limonene oxide	22.78	1137	1134	0.1	MS, RI
*trans*-Limonene oxide	23.05	1141	1138	0.1	MS, RI
Camphor	23.62	1150	1145	tr ^d^	MS, RI
(*E*)-2-Nonenal	24.28	1160	1162	0.1	MS, RI
Octanoic acid	25.25	1176	1180	0.1	MS, RI
Decanal	27.23	1207	1206	0.3	MS, RI
(*Z*)-3-Octenyl acetate	27.63	1213	1210	1.9	MS, RI
Fenchyl acetate	28.42	1225	1224	2.6	MS, RI
Methylthymol	29.23	1237	1235	0.1	MS, RI
(*E*)-2-Decenal	31.40	1271	1263	53.8	MS, RI
Bornyl acetate	32.77	1292	1285	1.0	MS, RI
*n*-Decanoic acid	37.91	1372	1372	0.3	MS, RI
(*E*)-2-Decenyl acetate	40.53	1414	1406	24.4	MS, RI
*trans*-2-Decenoic acid	41.18	1425	1428	2.2	MS, RI
(*E*)-2-Dodecenal	43.88	1469	1468	0.4	MS, RI
*β*-Selinene	45.52	1495	1486	0.1	MS, RI
(*Z*)-3-Dodecenyl acetate	51.13	1591	1591	3.5	MS, RI
Total (%)				97.0	
Yield (w/w, %)				0.56	

a Retention indices (RI) were determined utilizing n-alkanes (C_8_–C_16_). ^b^RI were obtained through NIST 2020 database. ^c^Identification: MS, comparing MS similarity with NIST 2020 and Wiley 275 databases; RI, comparison of calculated RI with those in NIST 2020 database. ^d^tr, trace < 0.1%.

### 3.2 Chemical composition of WE and EE

**Figure 2:**
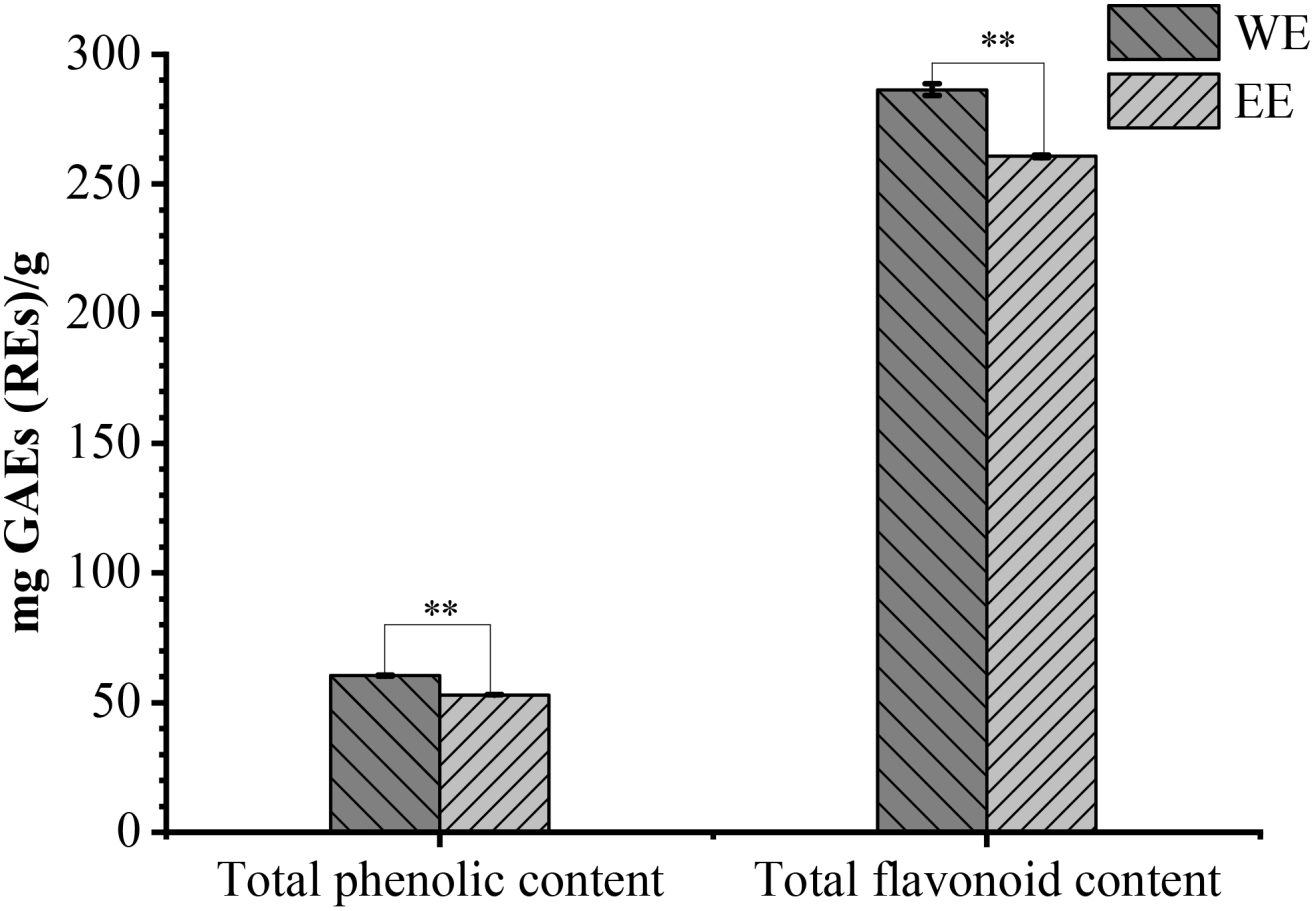
Total phenolic and flavonoid contents of *A. coriandriodora* rhizome WE and EE, **p < 0.01.

Based on the fresh weight of *A. coriandriodora* rhizome, the yield of WE and EE were 2.04% (w/w) and 1.37% (w/w), respectively. As shown in **Figure 2**, *A. coriandriodora* rhizome WE (60.49 ± 0.24 mg GAEs/g extract) exhibited more TPC compared to EE (52.99 ± 0.16 mg GAEs/g extract) (*p* < 0.01). The TFC in WE (286.42 ± 2.21 mg REs/g extract) was notably greater than that in EE (260.69 ± 0.44 mg REs/g extract) (*p* < 0.01).

The phytochemical components of WE and EE were further analyzed using UHPLC-Q-Orbitrap-MS. Sixty-three chemicals were identified based on comparisons of primary and secondary mass spectrometry with the mzVault and mzCloud databases and references (**Table 2**). As illustrated in **Figure S1** (**Supplementary Material**), WE and EE displayed variations in phytochemical constituents. Thirty-seven and fifty-three components were identified from WE and EE, respectively. WE and EE had high TPC and TFC values, and fourteen identified phenolic compounds and twenty-three flavonoid compounds were identified. The fourteen identified phenolic compounds included hordenine (5), protocatechuic acid (8), 3,4-dihydroxyphenylethanol (9), methyl gallate (10), vanillic acid (11), protocatechualdehyde (12), 4-methoxysalicylic acid (13), orsellinic acid (15), 2-hydroxy-4-methoxybenzaldehyde (19), *p*-coumaric acid (21), ethyl 3,4-dihydroxybenzoate (33), ferulaldehyde (34), tetrahydrocurcumin (36), and arctigenin (39). The twenty-three identified flavonoid compounds consisted of epicatechin (14), procyanidin B1 (16), astilbin (17), cianidanol (18), (+)-catechin hydrate (20), procyanidin A2 (23), hyperoside (24), quercetin (26), isoquercitrin (27), quercetin 3-*O*-*β*-*D*-Glucuronide (29), tetrahydroxyxanthone (31), morin (35), pectolinarigenin (40), lysionotin (41), isorhamnetin (43), isoxanthohumol (44), gardenin B (46), hydroxygenkwanin (47), 6-demethoxytangeretin (48), jaceosidin (49), eupatilin (50), 4’,7-di-*O*-methylnaringenin (53), and acacetin (57). All sixty-three constituents were first identified from *A. coriandriodora*. These findings suggested that *A. coriandriodora* rhizome can serve as a rich source of flavonoids and phenolic compounds.

**Table 2:** Phytochemical compounds of *A. coriandriodora* rhizome WE and EE detected and characterized using UHPLC-Q-Orbitrap-MS in positive and negative ionization modes.

Peak NO.	RT [min]	Identification^a^	Formula	[M+H]^+^(m/z)	[M-H]^-^(m/z)	Error ppm	MS^2^ fragment ions	WE^b^	EE^b^
1	0.712	Lactose	C_12_H_22_O_11_		387.11337[M+HCOO]^-^	-2.7	101.02293, 119.03342, 161.04428, 179.05481, 341.10782	√	–
2	0.714	Sucrose	C_12_H_22_O_11_		341.10797	-2.8	113.02293, 119.03345, 161.04437, 179.05476	√	√
3	1.214	Adenosine	C_10_H_13_N_5_O_4_	268.10339		-2.4	94.04030, 119.03508, 136.06160	√	√
4	1.248	L-Leucine	C_6_H_13_NO_2_	132.10176		-1.1	69.07053, 86.09682, 114.09131	√	√
5	1.470	Hordenine	C_10_H_15_NO	166.12222		-2.5	93.07013, 103.05432, 107.04924, 120.08076, 148.11159	√	–
6	1.884	8-*O*-Acetylharpagide	C_17_H_26_O_11_	424.17990 [M+NH_4_]^+^		-3.4	149.05927, 167.06981, 177.05402, 209.08041, 277.09064	–	√
7	2.047	L-Phenylalanine	C_9_H_11_NO_2_	166.08598		-1.7	91.05439, 103.05436, 120.08083	√	√
8	3.008	Protocatechuic acid	C_7_H_6_O_4_		153.01800	-8.7	91.01751, 108.02017, 109.02803, 153.01791	√	√
9	2.495	3,4-Dihydroxyphenylethanol	C_8_H_10_O_3_		153.05437	-8.8	91.01721, 108.02016, 109.02803, 123.04368	√	–
10	4.456	Methyl gallate	C_8_H_8_O_5_		183.02863	-6.9	107.01273, 123.00713, 124.01511, 139.03859, 168.02859	√	√
11	4.636	Vanillic acid	C_8_H_8_O_4_	169.04918		-2.1	93.03379, 111.04413, 123.04395, 125.05957, 151.03864	–	√
12	4.641	Protocatechualdehyde	C_7_H_6_O_3_		137.02290	-9.5	93.03308, 108.89825, 109.02795, 119.01244	√	√
13	7.236	4-Methoxysalicylic acid	C_8_H_8_O_4_		167.03365	-8.0	108.02039, 123.04395, 152.01016	–	√
14	7.958	Epicatechin	C_15_H_14_O_6_		289.07100	-0.7	125.02299, 137.02292, 203.07024, 227.07117, 245.08112	√	√
15	8.360	Orsellinic acid	C_8_H_8_O_4_	169.04913		-2.4	93.03359, 109.02838, 123.04395, 151.03860	–	√
16	8.757	Procyanidin B1	C_30_H_26_O_12_		577.13380	-2.3	125.02297, 289.07101, 407.07596, 425.08710, 451.10214	√	√
17	10.049	Astilbin	C_21_H_22_O_11_	451.12186		-3.6	123.04391, 135.04364, 139.03847, 151.03844, 163.03827	–	√
18	9.464	Cianidanol	C_15_H_14_O_6_	291.08539		-3.2	123.04387, 139.03856, 147.04355, 179.06952, 273.07553	√	–
19	10.122	2-Hydroxy-4-methoxybenzaldehyde	C_8_H_8_O_3_	153.05429		-2.2	93.03377, 111.04411, 125.05956, 135.11649	–	√
20	10.419	(+)-Catechin hydrate	C_15_H_16_O_7_		289.07100[M-H_2_O-H]^-^	-2.6	109.02806, 125.02304, 151.03873, 203.07028, 245.08105	√	√
21	10.921	*p*-Coumaric acid	C_9_H_8_O_3_		163.03874	-8.2	91.05386, 117.03326, 119.04878	√	√
22	10.928	Coumarin	C_9_H_6_O_2_	147.04366		-2.7	91.05455, 103.05450, 119.04910, 129.06928, 147.04362	√	√
23	12.457	Procyanidin A2	C_30_H_24_O_12_		575.11798	-2.6	109.02805, 125.02296, 285.03976, 289.07117, 449.08676	√	√
24	12.837	Hyperoside	C_21_H_20_O_12_		463.08710	-2.4	243.02907, 255.02910, 271.02414, 300.02667, 301.03455	√	√
25	12.578	Cafestol	C_20_H_28_O_3_	317.21014		-3.1	107.08553, 119.08534, 253.19479, 271.20447, 299.19955	√	-
26	12.839	Quercetin	C_15_H_10_O_7_	303.04910		-2.7	137.02304, 153.01773, 201.05397, 229.04869, 257.04324	√	√
27	13.003	Isoquercitrin	C_21_H_20_O_12_		463.08707	-2.4	151.00233, 255.02908, 271.02405, 300.02664, 301.03452	–	√
28	13.477	Azelaic acid	C_9_H_16_O_4_		187.09634	-6.6	97.06435, 125.09573, 143.10612, 169.08604	√	–
29	13.592	Quercetin 3-*O*-*β*-D-Glucuronide	C_21_H_18_O_13_		477.06625	-2.5	151.00233, 178.99590, 271.02405, 285.04007, 301.03458	√	–
30	14.621	Isoeugenol acetate	C_12_H_14_O_3_	207.10101		-2.7	91.05444, 115.05414, 147.07997, 175.07480, 192.07735	√	–
31	14.842	Tetrahydroxyxanthone	C_13_H_8_O_6_		259.02402	-3.0	109.02773, 151.00247, 215.03400, 231.02876	√	–
32	13.342	Benzoic acid	C_7_H_6_O_2_	123.04395		-0.9	77.03902, 95.04935, 105.03356	–	√
33	14.030	Ethyl 3,4-dihydroxybenzoate	C_9_H_10_O_4_		181.04936	-7.0	93.03310, 109.02799, 137.02245, 152.01010, 153.01790	–	√
34	14.621	Ferulaldehyde	C_10_H_10_O_3_	179.06981		-2.6	91.05450, 119.04903, 133.05450, 147.04362, 164.04637	√	√
35	16.319	Morin	C_15_H_10_O_7_	303.04886		-3.5	153.01776, 165.01814, 229.04861, 257.04370, 285.03806	√	√
36	17.530	Tetrahydrocurcumin	C_21_H_24_O_6_	373.16354		-2.7	131.04883, 137.05934, 163.07486, 177.05409, 193.05514	√	√
37	17.678	Aconitine	C_34_H_47_NO_11_	646.31989		-3.6	105.03362, 368.18655, 526.27710, 554.27283, 586.30042	–	√
38	17.740	Dendrobine	C_16_H_25_NO2	264.19501		-3.0	119.07289, 190.15697, 204.17429, 218.18962, 246.18449	–	√
39	18.947	Arctigenin	C_21_H_24_O_6_	373.16318		-3.7	123.04391, 163.07481, 167.05999, 235.10721, 355.23544	–	√
40	19.036	Pectolinarigenin	C_17_H_14_O_6_	315.08508		-3.9	121.06457, 167.03343, 244.07216, 272.05689, 300.06158	√	√
41	19.467	Lysionotin	C_18_H_16_O_7_	345.09600		-2.6	92.02620, 287.05386, 301.06958, 315.04889, 330.0211	–	√
42	19.518	Ingenol	C_20_H_28_O_5_		347.18533	-3.1	257.19089, 285.18518, 303.19473, 317.17480, 329.17480	√	√
43	20.190	Isorhamnetin	C_16_H_12_O_7_		315.05020	-2.6	121.02800, 151.00209, 163.00255, 165.01796, 271.02475, 300.02676	√	√
44	20.977	Isoxanthohumol	C_21_H_22_O_5_	355.15274		-3.5	94.04169, 137.05940, 177.08997, 219.10103	√	√
45	21.388	*o*-Veratraldehyde	C_9_H_10_O_3_	167.06960		-4.0	95.04935, 109.02849, 123.0435, 137.02356, 152.04628	√	√
46	21.556	Gardenin B	C_19_H_18_O_7_	359.11142		-3.1	227.06918, 301.06961, 329.06430, 344.08755	–	√
47	22.376	Hydroxygenkwanin	C_16_H_12_O_6_		299.05530	-2.7	93.03309, 107.01242, 151.00223, 165.01802, 284.03174	√	√
48	22.385	6-Demethoxytangeretin	C_19_H_18_O_6_	343.11660		-2.9	211.07457, 254.09286, 282.08759, 310.08234, 327.08469	√	–
49	22.721	Jaceosidin	C_17_H_14_O_7_	331.08023		-3.0	93.03390, 151.03886, 167.03339, 299.05319, 316.05655	√	√
50	23.370	Eupatilin	C_18_H_16_O_7_	345.09589		-2.9	151.03835, 287.05374, 315.04858, 330.07184	√	√
51	25.704	Camphor	C_10_H_16_O	153.12703		-2.4	69.07043, 93.07010, 97.06496, 107.08556, 135.11644	–	√
52	26.288	Atractylenolide I	C_15_H_18_O_2_	231.13737		-2.6	95.04933, 119.08540, 173.09563, 185.13188, 213.12668	–	√
53	26.376	4’,7-Di-*O*-methylnaringenin	C_17_H_16_O_5_	301.10620		-2.8	118.04107, 133.06444, 161.05919, 167.03337	–	√
54	26.736	Dehydrocostus lactone	C_15_H_18_O_2_	231.13730		-2.9	175.07486, 185.13152, 189.12680, 203.14258, 213.12672	–	√
55	27.649	*α*-Cyperone	C_15_H_22_O	219.17361		-3.3	147.11630, 159.11624, 177.12659, 191.17879, 201.16316	–	√
56	28.068	(+)-Nootkatone	C_15_H_22_O	219.17392		-1.9	93.07009, 121.10107, 135.08000, 163.11107, 201.16293	√	√
57	28.558	Acacetin	C_16_H_12_O_5_	285.07480		-3.3	135.04379, 14.06790, 241.04897, 242.05641, 270.05063	–	√
58	29.636	Germacrone	C_15_H_22_O	219.17375		-2.7	95.08570, 121.10097, 137.09563, 159.11624, 201.16298	–	√
59	31.321	Kahweol	C_20_H_26_O_3_	315.19461		-2.7	123.11662, 45.10086, 159.11626, 173.13190, 175.14748	–	√
60	34.529	Abietic Acid	C_20_H_30_O_2_	303.23100		-2.8	93.07008, 107.08556, 109.10124, 121.10103, 217.19438	–	√
61	36.199	Linolenic acid ethyl ester	C_20_H_34_O_2_	307.26199		-3.8	79.05461, 95.08575, 113.09608, 161.13188, 179.14244	–	√
62	38.742	*α*-Linolenic acid	C_18_H_30_O_2_	279.23096		-3.2	67.05480, 79.05468, 109.10131, 123.04386, 191.14226	–	√
63	40.834	Methyl linoleate	C_19_H_34_O_2_	295.26215		-3.4	67.05481, 81.07029, 95.08569, 109.10120, 169.12172	–	√

a Identification: Based on comparison with mzCloud and mzVault databases and references. ^b^“√” means detected from extracts, “-” means undetected from extracts.

### 3.3 Antioxidant capacity

In the antioxidant capacity of *A. coriandriodora* rhizome (**Table 3**), the scavenging effect of EO on DPPH and ABTS was weak. WE (IC_50_: 6.59 ± 0.88 μg/mL) and EE (IC_50_: 17.70 ± 1.15 μg/mL) possessed potent DPPH scavenging ability, which was superior to that of BHT (IC_50_: 21.83 ± 0.89 μg/mL) (*p* < 0.05). In the ABTS analysis, WE and EE exhibited significant ABTS radical scavenging activity, with IC_50_ values of 20.48 ± 0.44 μg/mL and 18.27 ± 0.55 μg/mL, respectively. Excessive production of reactive oxygen species triggers oxidative stress, which could induce or aggravate many chronic diseases, such as neurodegenerative disorders, cardiovascular disease, asthma, and cancer (López-Alarcón and Denicola, 2013; Pisoschi and Pop, 2015). Past studies have revealed that various phenolic and flavonoid compounds from *Alpinia* genus plants were natural antioxidants that are effective in scavenging free radicals (Honmore et al., 2016; Tang et al., 2018; da Cruz et al., 2020). Based on the results of UHPLC-Q-Orbitrap-MS, a variety of identified phenolic and flavonoid compounds, such as protocatechuic acid (8), methyl gallate (10), vanillic acid (11), and epicatechin (14), has been proved to have DPPH and ABTS scavenging effects (Kakkar and Bais, 2014; Li et al., 2014; Bernal-Mercado et al., 2018; Ryu and Kim, 2022). Hence, the antioxidant activity of *A. coriandriodora* rhizome WE and EE can be attributed to their richness in phenolics and flavonoids, which can serve as a natural source of antioxidants.

**Table 3:** Antioxidant activity of EO, WE and EE from *A. coriandriodora* rhizome.

Treatment	DPPH		ABTS		Yield (W/W)
	IC_50_ (μg/mL) ^1^	mg BEs/g sample ^2^	IC_50_ (μg/mL) ^1^	mg BEs/g sample ^2^	
EO	>1000	–	>1000	–	0.56%
WE	6.59 ± 0.88 ^a^	3342.6 ± 352.46 ^a^	20.48 ± 0.44 ^a^	308.72 ± 18.40 ^a^	2.04%
EE	17.70 ± 1.15 ^b^	1238.4 ± 123.88 ^b^	18.27 ± 0.55 ^b^	346.10 ± 18.16 ^b^	1.37%
BHT ^3^	21.83 ± 0.89 ^c^		6.32 ± 0.24 ^c^		

^1^IC_50_: The concentration of sample that scavenged 50% free radical. ^2^The mg BEs/g sample represents milligrams of BHT equivalent per gram of sample. ^3^BHT as positive control. ^a-c^Different letters in the same column represent significant difference (p < 0.05).

### 3.4 Antibacterial properties

The antibacterial properties of EO, WE, and EE were determined qualitatively using the DIZ and assessed quantitatively using the MIC and MBC. Streptomycin was used as the control drug (**Table 4**). The DIZ of WE and EE (100 mg/mL) were not observed, and the MIC and MBC of WE and EE were not listed as they were not detected at 25 mg/mL. EO exhibited broad-spectrum antibacterial activity with DIZ values ranging from 12.60 to 22.17 mm. Previous studies suggest that MIC values less than 5 mg/mL have potent antibacterial properties (Monteiro et al., 2021). Hence, the EO displayed a strong antibacterial effect against *P. vulgaris* (MIC = 1.56 mg/mL, MBC = 3.13 mg/mL), *E. faecalis* (MIC = 0.78 mg/mL, MBC = 3.13 mg/mL), *B. subtilis* (MIC = 0.78 mg/mL, MBC = 3.13 mg/mL), *E. coli* (MIC = 0.78 mg/mL, MBC = 6.25 mg/mL) and *S. aureus* (MIC = 0.78 mg/mL, MBC = 3.13 mg/mL). In addition, it revealed moderate antibacterial activity against *P. aeruginosa* (MIC = 6.25 mg/mL, MBC = 6.25 mg/mL). (*E*)-2-Decenal, as the predominant component, has been proven to possess broad-spectrum antibacterial action, with MIC values ranging from 7.8 to 500 μg/mL against *E. coli*, *S. aureus*, *S. epidermidis*, *Salmonella typhi*, *S. enteritidis*, *Bacillus cereus*, *Moraxella catarrhalis*, *Haemophilus influenzae*, *Listeria monocytogenes*, *Streptococcus pneumoniae*, and *S. pyogenes* (Bisignano et al., 2001; Trombetta et al., 2002). (*E*)-2-Octenal was a potential antibacterial agent that inhibited the growth of a variety of bacteria, including *S. aureus*, *E. coli, P. vulgaris*, *P. aeruginosa, Enterobacter aerogenes*, *Propionibacterium acnes*, *Streptococcus mutans*, *Brevibacterium ammoniagenes*, and *Bacillus subtilis* (Kubo and Kubo, 1995; Sagun et al., 2016). Thus, the antibacterial action of *A. coriandriodora* EO can be attributed to these main components. Bacterial infections and the emergence of bacterial resistance have threatened human public health around the world (Fernandes et al., 2022). Therefore, EO with antibacterial properties has gained great attention. Our results suggested that *A. coriandriodora* EO may be used in the food and pharmaceutical industries as a natural antibacterial agent.

**Table 4:** Antibacterial properties of *A. coriandriodora* rhizome EO, WE, and EE.

Bacterial strains ^a^	EO			Streptomycin		
	DIZ ^b^ (mm)	MIC ^c^ (mg/mL)	MBC ^c^ (mg/mL)	DIZ ^b^ (mm)	MIC ^c^ (μg/mL)	MBC ^c^ (μg/mL)
Gram-positive						
*E. faecalis*	15.59 ± 0.25	0.78	3.13	10.22 ± 0.79	0.78	12.5
*S. aureus*	22.17 ± 0.89	0.78	3.13	25.00 ± 0.33	3.13	3.125
*B. subtilis*	17.74 ± 1.00	0.78	3.13	32.06 ± 1.07	3.13	3.125
Gram-negative						
*P. aeruginosa*	12.60 ± 0.53	6.25	6.25	10.11 ± 0.31	6.25	6.25
*E. coli*	14.93 ± 0.65	0.78	6.25	19.08 ± 0.85	3.13	12.5
*P. vulgaris*	13.67 ± 0.68	1.56	3.13	18.35 ± 0.49	3.13	12.5

a Bacterial strains: Staphylococcus aureus (ATCC 6538P), Bacillus subtilis (ATCC 6633), Enterococcus faecalis (ATCC 19433), Pseudomonas aeruginosa (ATCC 9027), Escherichia coli (CICC 10389), and Proteus vulgaris (ACCC 11002). ^b^DIZ, diameter of inhibition zone (mm, including 6 mm disk). Disks contained 20 µL of EO (100 mg/mL) or streptomycin (100 µg/mL). ^c^MIC, minimal inhibitory concentration; MBC, minimal bactericidal concentration. The DIZ of WE and EE (100 mg/mL) were not observed. The MIC and MBC of WE and EE were not listed as they were not detected at 25 mg/mL.

### 3.5 Antifungal capacity

**Figure 3:**
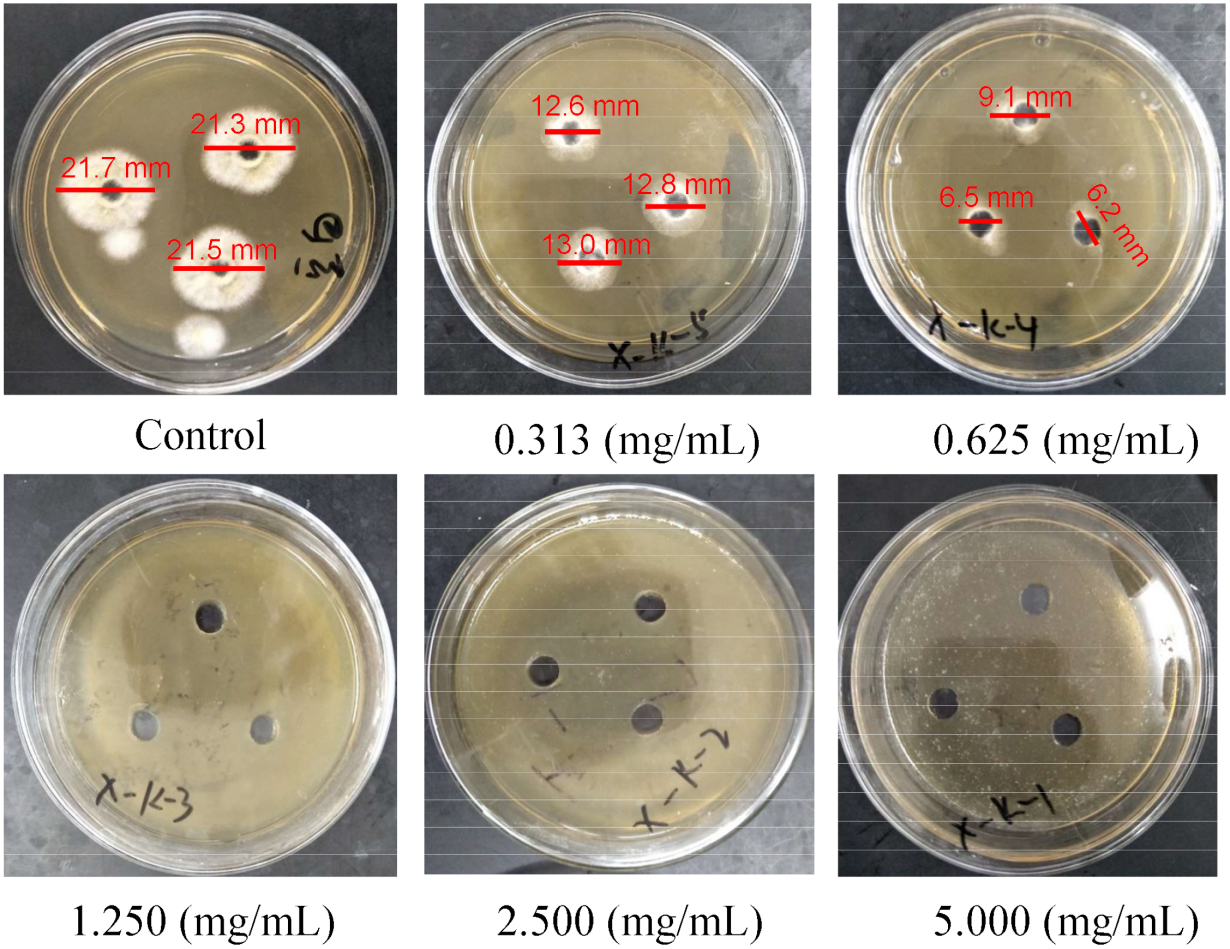
Anti-*Aspergillus flavus* activity of *A. coriandriodora* rhizome EO.

The anti-*Aspergillus flavus* properties of *A. coriandriodora* rhizome EO are shown in **Figure 3** and **Table 5**. The EO exhibited anti-*Aspergillus flavus* effects in a dose-dependent manner. The growth of *A. flavus* on the PDA medium was obviously inhibited when the EO concentration reached 0.625 mg/mL (MIC). Besides, EO completely inhibited *A. flavus* growth at the concentration of 1.250 mg/mL (MFC). (*E*)-2-Decenal is a potent antifungal agent against several fungi, including *Trichophyton mentagrophytes*, *Penicillium chrysogenum*, *Pityrosprum ovale*, *Candida utilis*, *Saccharomyces cerevisiae*, *Purpureocillium lilacinum*, *Isaria fumosorosea*, *Metarhizium flavoviride*, *Paecilomyces suffultus*, *Beauveria bassiana*, and *Akanthomyces lecanii* (Kubo and Kubo, 1995; Bojke et al., 2020). (*E*)-2-Octenal has been proven to be a substitute for chemical fungicides with antifungal activity against *Trichophyton mentagrophytes*, *Penicillium italicum*, *Sclerotium rolfsii*, *Aspergillus niger*, *Penicillium digitatum*, *Alternaria alternate*, *Microsporum canis*, *Botrytis cinerea*, etc. (Battinelli et al., 2006; Liarzi et al., 2016; Liarzi et al., 2020). In particular, (*E*)-2-octenal exerted a potent inhibitory effect on the growth of *A. flavus* and the production of aflatoxin B1 (Cleveland et al., 2009). Hence, these compounds could explain the remarkable anti-*Aspergillus flavus* activity of *A. coriandriodora* rhizome EO. *A. flavus* is known to cause spoilage of vegetables, grains, fruits, and nuts, which results in huge economic losses worldwide (Hashem et al., 2022). In addition, aflatoxin produced by *A. flavus* poses a great threat to human health due to its immunosuppressive, genotoxic, and carcinogenic effects (Lasram et al., 2019; Yadav et al., 2019). Recent studies have suggested that essential oils can be used as a greener alternative to protect foods from *Aspergillus flavus* contamination (Dutta et al., 2020; Kundu et al., 2022). Hence, *A. coriandriodora* rhizome EO has the exploitation potential as an anti-*Aspergillus flavus* agent in the food field.

**Table 5:** Anti-*Aspergillus flavus* properties of *A. coriandriodora* rhizome EO.

Bacterial strains	EO		Amphotericin B	
	MIC (mg/mL)	MFC (mg/mL)	MIC (μg/mL)	MFC (μg/mL)
*Aspergillus flavus* (AS3.3950)	0.625	1.25	6.25	6.25

### 3.6 Enzyme inhibitory properties

The enzyme inhibitory properties of *A. coriandriodora* rhizome WE, EE, and EO on cholinesterases (AChE and BChE), tyrosinase, and α-glucosidase were tested, and the results are shown in **Table 6**.

**Table 6:** The enzyme inhibitory properties of *A. coriandriodora* rhizome EO, WE, and EE.

Samples	Acetylcholinesterase		Butyrylcholinesterase		Tyrosinase		α-Glucosidase	
	IC_50_ (mg/mL)	(mg GALAEs/g sample)	IC_50_ (mg/mL)	(mg GALAEs/g sample)	IC_50_ (mg/mL)	(mg AREs/g sample)	IC_50_ (mg/mL)	(mmoL ACEs/g sample)
EO	5.84 ± 0.48 ^a^	0.045 ± 0.004 ^a^	5.73 ± 0.33 ^a^	1.44 ± 0.07 ^a^	1.01 ± 0.08 ^a^	253.12 ± 9.13 ^a^	3.30 ± 0.17 ^a^	0.11 ± 0.01 ^a^
WE	6.67 ± 0.04 ^a^	0.039 ± 0.001 ^a^	3.61 ± 0.89 ^b^	2.40 ± 0.70 ^a^	8.91 ± 1.69 ^b^	29.78 ± 5.61 ^b^	0.017 ± 0.002 ^b^	19.92 ± 1.19 ^b^
EE	1.59 ± 0.09 ^b^	0.16 ± 0.02 ^b^	0.38 ± 0.02 ^c^	21.74 ± 1.46 ^b^	19.85 ± 3.74 ^c^	13.21 ± 2.71 ^c^	0.013 ± 0.001 c	25.87 ± 2.32 c
Galanthamin*	0.26 ± 0.01 ^c^		8.23 ± 0.14 ^d^					
Arbutin					0.26 ± 0.01 ^d^			
Acarbose							0.22 ± 0.01 ^d^	

IC_50_: The concentration of sample that affords a 50% inhibition in the assay. The mg GALAEs/g sample represents milligrams of galanthamine equivalent per gram of sample; The mg AREs/g sample refers to milligrams of arbutin equivalent per gram of sample; The mmoL ACEs/g sample means mmoL of acarbose equivalent per gram of sample. ^a-d^ Different letters in the same column represent significant difference (p<0.05). *Galanthamine: IC_50_ (μg/mL).

AD (Alzheimer’s disease) is a chronic disease with memory loss and cognitive impairment caused by central nervous system degeneration, and low levels of cholinergic energy in the human brain play an essential role in the pathogenesis of AD (Sharma, 2019). As shown in **Table 6**, compared with the positive control galanthamine, EO, WE, and EE of *A. coriandriodora* rhizome showed weak AChE (IC_50_: 5.84 ± 0.48 mg/mL, 6.67 ± 0.04 mg/mL, and 1.59 ± 0.09 mg/mL, respectively) and BChE (IC_50_: 5.73 ± 0.33 mg/mL, 3.61 ± 0.89 mg/mL, and 0.38 ± 0.02 mg/mL, respectively) inhibitory activity.

In mammals, tyrosinase is a crucial enzyme in the production of melanin, which protects against UV damage, but excessive melanin can lead to many skin problems, such as malignant melanoma, freckles, and age spots (Brenner and Hearing, 2008; Sun et al., 2017). Tyrosinase inhibitors have been extensively applied in cosmetic and medicine industries to solve these problems. According to the results presented in **Table 6**, EO (IC_50_: 1.01 ± 0.08 mg/mL, 253.12 ± 9.13 mg AREs/g sample) had the most strong tyrosinase inhibition, followed by WE (IC_50_: 8.91 ± 1.69 mg/mL, 29.78 ± 5.61 mg AREs/g sample) and EE (IC_50_: 19.85 ± 3.74 mg/mL, 13.21 ± 2.71 mg AREs/g sample). (*E*)-2-octenal, as the main components of *A. coriandriodora* rhizome EO, have been demonstrated to possess tyrosinase inhibition (Kubo and Kinst-Hori, 1999). Plant-derived flavonoids and phenolic compounds can serve as natural inhibitors of tyrosinase (Goncçalves and Romano, 2017). A variety of phenolic and flavonoid compounds identified from WE and EE, such as orsellinic acid, arctigenin, and hordenine, have been confirmed to possess tyrosinase inhibitory activity (Kim et al., 2013; Park et al., 2013; Lopes et al., 2018). Thus, the tyrosinase inhibition of *A. coriandriodora* rhizome can be attributed to the presence of these active ingredients.

The α-glucosidase inhibitors can decrease the intake of postprandial carbohydrates by reducing the digestion of carbohydrates and are an effective means of treating type II diabetes (Gao et al., 2022). Nevertheless, synthetic α-glucosidase inhibitors, such as voglibose, emiglitate, miglitol, and acarbose, have unacceptable consequences, including flatulence, nausea, vomiting, diarrhea, stomach pain, and allergic reactions (Liu et al., 2015; Hossain et al., 2020). Hence, there is an urgent need to find more effective and safer natural products as α-glucosidase inhibitors. As illustrated in **Table 6**, the inhibitory effects of *A. coriandriodora* rhizome on α-glucosidase were as follows: EE (IC_50_: 0.013 ± 0.001 mg/mL) > WE (IC_50_: 0.017 ± 0.002 mg/mL) > acarbose (IC_50_: 0.22 ± 0.01 mg/mL) > EO (IC_50_: 3.30 ± 0.17 mg/mL) (*p* < 0.05). Notably, the α-glucosidase inhibitory capacity of WE (19.92 ± 1.19 mmoL ACEs/g sample) and EE (25.87 ± 2.32 mmoL ACEs/g sample) was significantly higher than acarbose (*p* < 0.05).

Previous studies have indicated that phenolic compounds and flavonoids can act as potent α-glucosidase inhibitors (Goncçalves and Romano, 2017). Various phenolic and flavonoid compounds identified from *A. coriandriodora* rhizome, such as hordenine (Su et al., 2018), *p*-coumaric acid (Amalan et al., 2016), procyanidin A_2_ (Sheikh et al., 2019), eupatilin (Kang et al., 2008), and acacetin (Wang et al., 2023) were obviously lowered fasting blood glucose level of diabetic mice. In addition, protocatechualdehyde (Ji et al., 2021; Luo et al., 2021), hyperoside (Ku et al., 2014; Luo et al., 2021), quercetin (Vessal et al., 2003; Nurul Islam et al., 2013), and isoquercitrin (Zhang et al., 2018; Luo et al., 2021) displayed significant anti-diabetic properties *in vivo* and α-glucosidase inhibitory properties *in vitro* that were stronger than the positive control acarbose. Furthermore, epicatechin (Lin et al., 2022) and pectolinarigenin (Amin et al., 2017) showed stronger inhibitory efficacy against α-glucosidase compared to acarbose. These phenolic and flavonoid compounds may contribute to the potent α-glucosidase inhibition of *A. coriandriodora* rhizome WE and EE. Hence, *A. coriandriodora* rhizome WE and EE could be utilized as a novel source of α-glucosidase inhibitors for the functional food and pharmaceutical industries.

## 4 Conclusions

In addition to the chemical composition of *A. coriandriodora* rhizome EO, this study is the first to analyze the phytochemical components of WE and EE and evaluate their antioxidant, antimicrobial, and enzyme inhibitory properties. According to the GC-MS data, the primary components of *A. coriandriodora* rhizome EO were (*E*)-2-decenal, (*E*)-2-decenyl acetate, (*Z*)-3-dodecenyl acetate, and (*E*)-2-octenal. *A. coriandriodora* rhizome WE and EE were abundant in phenolics and flavonoids. UHPLC-Q-Orbitrap-MS analysis further identified sixty-three compounds in WE and EE, including 14 phenolic compounds and 23 flavonoids. WE and EE exhibited a potent DPPH radical scavenging effect, superior to the positive control BHT. Besides, EO displayed powerful antimicrobial activity against *E. faecalis*, *S. aureus*, *B. subtilis*, *E. coli*, *P. vulgaris*, and *A. flavus.* In enzyme inhibitory activity, the EE and WE revealed a remarkable α-glucosidase inhibition, which were higher than positive control acarbose. Hence, *A. coriandriodora* rhizome may be used as a source of bioactive substances and has excellent potential for exploitation in the pharmaceutical and food fields.

## Data availability statement

The datasets presented in this study can be found in online repositories. The names of the repository/repositories and accession number(s) can be found below: MetaboLights, MTBLS8631.

## Author contributions

XW: Investigation, Methodology, Writing – original draft. FW: Investigation, Validation, Writing – original draft. FD: Investigation, Validation, Writing – original draft. NY: Investigation, Validation, Writing – original draft. JN: Investigation, Writing – original draft. YR: Formal Analysis, Writing – original draft. MT: Conceptualization, Funding acquisition, Methodology, Supervision, Validation, Writing – original draft, Writing – review & editing.
