# Supplementary material for: Phytochemical analysis, antioxidant, antimicrobial, and anti-enzymatic properties of Alpinia coriandriodora (sweet ginger) rhizome
Source: Front Plant Sci. 2023 Oct 23;14:1284931. doi: 10.3389/fpls.2023.1284931 (PMC10626549; doi:10.3389/fpls.2023.1284931)
Supplement: Supplementary file 1 [file DataSheet_1.docx]

**Supplementary Data**

**FIGURE S1.** UHPLC-Q-Orbitrap-MS base peak chromatograms of *Alpinia coriandriodora* rhizome water extract (WE) and 70% ethanol extract (EE). **(A)** EE in positive ion mode; **(B)** EE in negative ion mode; **(C)** WE in positive ion mode; **(D)** WE in negative ion mode.

[1] Lactose (Valgimigli et al., 2012)

[2] Sucrose (Valgimigli et al., 2012)

[3] Adenosine (Jiang et al., 2020)

[4] *L*-Leucine (Jiang et al., 2020)

[5] Hordenine (Tao, 2021)

[6] 8-*O*-Acetylharpagide (Taylor et al., 2005)

[7] L-Phenylalanine (Scheubert et al., 2013)

[8] Protocatechuic acid (Gutierrez-Zetina et al., 2019)

 [9] 3,4-Dihydroxyphenylethanol

[10] Methyl gallate (Shahzad et al., 2022)

[11] Vanillic acid (Ostrowski et al., 2014)

[12] Protocatechualdehyde (Huang et al., 2020; Kong et al., 2020)

[13] 4-Methoxysalicylic acid

[14] Epicatechin (Xu et al., 2021)

[15] Orsellinic acid (Ma et al., 2016)

[16] Procyanidin B1 (Heger et al., 2022; Weinert et al., 2012; Callemien and Collin, 2008)

[17] Astilbin (He et al., 2018)

[18] Cianidanol (Tao, 2021)

[19] 2-Hydroxy-4-methoxybenzaldehyde (Ding et al., 2019)

[20] (+)-Catechin hydrate (Xu et al., 2021)

 [21] *p*-Coumaric acid (Sinosaki et al., 2020; Yang et al., 2020)

[22] Coumarin (Ren et al., 2016; Concannon et al., 2000)

[23] Procyanidin A2 (Lv et al., 2022; Wong-Paz et al., 2021)

[24] Hyperoside (Ji et al., 2022)

[25] Cafestol (Andriolo et al., 2021; Dias et al., 2013)

[26] Quercetin (Scigelova et al., 2011; Ma et al., 2022)

[27] Isoquercitrin (Wang, 2015)

[28] Azelaic acid (Abu-Reidah et al., 2013)

[29] Quercetin 3-O-β-D-Glucuronide (Huang et al., 2020; Scigelova et al., 2011)

[30] Isoeugenol acetate (Gadiwiyana et al., 2019)

[31] Tetrahydroxyxanthone

[32] Benzoic acid (Jia et al., 2023)

[33] Ethyl 3,4-dihydroxybenzoate (Sinosaki et al., 2020)

[34] Ferulaldehyde

[35] Morin (Scigelova et al., 2011)

[36] Tetrahydrocurcumin (Shi et al., 2019; Tan et al., 2014)

[37] Aconitine (Zhang et al., 2012)

[38] Dendrobine (Pan et al., 2022; Lu et al., 2022)

[39] Arctigenin (Liu et al., 2015; Boldizsár et al., 2010)

[40] Pectolinarigenin (Zhang et al., 2018)

[41] Lysionotin

[42] Ingenol

[43] Isorhamnetin (Justesen, 2000)

[44] Isoxanthohumol (Nikolic et al., 2005)

 [45] *o*-Veratraldehyde

[46] Gardenin B (Jia et al., 2023)

 [47] Hydroxygenkwanin (Yuan et al., 2018)

[48] 6-Demethoxytangeretin (Fu et al., 2020; Zheng et al., 2020)

[49] Jaceosidin

[50] Eupatilin (Bojilov et al., 2017)

 [51] Camphor (García Martínez et al., 1999; Teso Vilar et al., 2011)

[52] Atractylenolide I (Zhang et al., 2019)

[53] 4',7-Di-*O*-methylnaringenin

[54] Dehydrocostus lactone (Tian et al., 2022)

[55] *α*-Cyperone (Syahbirin et al., 2020)

[56] (+)-Nootkatone (Li et al., 2016)

[57] Acacetin (Feng et al., 2014; Yin et al., 2019)

[58] Germacrone (Ying et al., 2021)

[59] Kahweol (Dias et al., 2014; Zhang et al., 2022)

[60] Abietic Acid (Rontani et al., 2015)

[61] Linolenic acid ethyl ester (Aparamarta et al., 2018)

[62] *α*-Linolenic acid (Jia et al., 2023)

[63] Methyl linoleate

**REFERENCES**

Abu-Reidah, I. M., Contreras, M. M., Arráez-Román, D., Segura-Carretero, A., and Fernández-Gutiérrez, A. (2013). Reversed-phase ultra-high-performance liquid chromatography coupled to electrospray ionization-quadrupole-time-of-flight mass spectrometry as a powerful tool for metabolic profiling of vegetables: *Lactuca sativa* as an example of its application. *J. Chromatogr.* 1313, 212–227. doi: 10.1016/j.chroma.2013.07.020

Andriolo, C. V., Novaes, F. J. M., Pereira, H. M. G., Sardela, V. F., and Rezende, C. M. (2021). Metabolic study of cafestol using *in silico* approach, zebrafish water tank experiments and liquid chromatography high-resolution mass spectrometry analyses. *J. Chromatogr. B* 1186, 123028. doi: 10.1016/j.jchromb.2021.123028

Aparamarta, H. W., Qadariyah, L., Gunawan, S., and Ju, Y. H. (2018). Separation and identification of fatty acid in triacyglycerol isolated from *Calophyllum inophyllum* oil. *Lipids* 70, 0–32. doi: 10.1063/1.4982284

Bojilov, D., Dagnon, S., and Ivanov, I. (2017). New insight into the flavonoid composition of *Chenopodium botrys. Phytochem. Lett.* 20, 316–321. doi: 10.1016/j.phytol.2017.01.015

Boldizsár, I., Füzfai, Z., Tóth, F., Sedlák, É., Borsodi, L., and Molnár-Perl, I. (2010). Mass fragmentation study of the trimethylsilyl derivatives of arctiin, matairesinoside, arctigenin, phylligenin, matairesinol, pinoresinol and methylarctigenin: Their gas and liquid chromatographic analysis in plant extracts. *J. Chromatogr. A* 1217, 1674–1682. doi: 10.1016/j.chroma.2010.01.019

Callemien, D., and Collin, S. (2008). Use of RP-HPLC-ESI(–)-MS/MS to Differentiate Various Proanthocyanidin Isomers in Lager Beer Extracts. *J. Am. Soc. Brew. Chem.* 66, 109–115. doi: 10.1094/ASBCJ-2008-0215-01

Concannon, S., Ramachandran, V. N., and Smyth, W. F. (2000). A study of the electrospray ionisation of selected coumarin derivatives and their subsequent fragmentation using an ion trap mass spectrometer. *Rapid Commun. Mass Spectrom.* 14, 1157–1166. doi: 10.1002/1097–0231(20000730)14:14<1157::AID-RCM4>3.0.CO;2-V

Ding, F., Liu, J., Du, R., Yu, Q., Gong, L., Jiang, H., et al. (2019). Qualitative and Quantitative Analysis for the Chemical Constituents of *Tetrastigma hemsleyanum* Diels et Gilg Using Ultra-High Performance Liquid Chromatography/Hybrid Quadrupole-Orbitrap Mass Spectrometry and Preliminary Screening for Anti-Influenza Virus Components. *Evid. Based Complement. Alternat. Med*. 2019, 9414926. doi: 10.1155/2019/9414926

Dias, R. C. E., de Faria-Machado, A. F., Mercadante, A. Z., Bragagnolo, N., and de Toledo Benassi, M. (2014). Roasting process affects the profile of diterpenes in coffee. *Eur. Food Res. Technol.* 239, 961–970. doi: 10.1007/s00217-014-2293-x

Dias, R. C., Faria, A. F. D., Mercadante, A. Z., Bragagnolo, N., Benassi, M. D. T. (2013). Comparison of extraction methods for kahweol and cafestol analysis in roasted coffee. *J. Braz. Chem. Soc*. 24, 492–499. doi: 0.5935/0103-5053.20130057

Fu, C., Liu, M., Li, Y., Wang, K., Yang, B., Deng, L., et al. (2020). UPLC-Q-exactive orbitrap MS analysis for identification of lipophilic components in Citri Sarcodactylis Fructus from different origins in China using supercritical CO_2_ fluid extraction method. *Acs Omega* 5, 11013–11023. doi: 10.1021/acsomega.0c00854

Feng, X., Liu, Y., Wang, X., and Di, X. (2014). Analysis of Linarin and Its Metabolites in Rat Urine by LC–MS/MS. *Chromatographia* 77, 571–579. doi: 10.1007/s10337-014-2641-9

García Martínez, A., Teso Vilar, E., García Fraile, A., de la Moya Cerero, S., and Martínez Ruiz, P. (1999). Electron impact fragmentation patterns of 3,3-dimethyl-1,2-norbornane derivatives. *Rapid Commun. Mass Spectrom*. 13, 1472–1476. doi: 10.1002/(SICI)1097-0231(19990730)13:14<1472::AID-RCM669>3.0.CO;2-V

Gadiwiyana, N., Ismiyarto, Gunawan, Sarjono, P. R., Adiwibawa Prasetya, N. B., Kusworo, T. D., et al. (2019). One pot reaction to synthesize allyl etherified eugenol from clove oil. *Iop Conf. Ser. Mater. Sci. Eng.* 509, 12098. doi: 10.1088/1757-899X/509/1/012098

Gutierrez-Zetina, S., Gonzalez-Manzano, S., Perez-Alonso, J., Gonzalez-Paramas, A., and Santos-Buelga, C. (2019). Preparation and Characterization of Protocatechuic Acid Sulfates. *Molecules* 24, 307. doi: 10.3390/molecules24020307

Huang, G., Liang, J., Chen, X., Lin, J., Wei, J., Huang, D., et al. (2020). Isolation and Identification of Chemical Constituents from Zhideke Granules by Ultra-Performance Liquid Chromatography Coupled with Mass Spectrometry. *J. Anal. Methods Chem*. 2020, 8889607. doi: 10.1155/2020/8889607

Heger, T., Zatloukal, M., Kubala, M., Strnad, M., and Gruz, J. (2022).  Procyanidin C1 from *Viola odorata* L. inhibits Na+,K+-ATPase. *Sci. Rep.* 12, 7011. doi: 0.1038/s41598-022-11086-y

He, L., Zhang, Z., Liu, Y., Chen, D., Yuan, M., Dong, G., et al. (2018). Rapid discrimination of raw and sulfur-fumigated *Smilax glabra* based on chemical profiles by UHPLC-QTOF-MS/MS coupled with multivariate statistical analysis. *Food Res. Int.* 108, 226–236. doi: 10.1016/j.foodres.2018.03.047

Jia, X., Yang, Y., Wang, Q., Tian, Y., Hong, Y., Tian, M., and Tang, D. (2023). Phytochemical composition, antioxidant, anti-tyrosinase, anti-cholinesterase, and anti-inflammatory activities of *Rhynchanthus beesianus* rhizome extracts. *Arab. J. Chem.* 16, 104952. doi: 10.1016/j.arabjc.2023.104952

Ji, L., Shi, W., Li, Y., He, J., Xu, G., Qin, M., et al. (2022). Systematic Identification, Fragmentation Pattern, And Metabolic Pathways of Hyperoside in Rat Plasma, Urine, And Feces by UPLC-Q-Exactive Orbitrap MS. *J. Anal. Methods. Chem.* 2022, 2623018. doi: 10.1155/2022/2623018

Justesen, U. (2000). Collision-induced fragmentation of deprotonated methoxylated flavonoids, obtained by electrospray ionization mass spectrometry. *J. Mass Spectrom.* 36, 169–178. doi: 10.1002/jms.118

Jiang, C., Arthur, C. J., and Gates, P. J. (2020). A computational and experimental study of the fragmentation of L-Leucine, L-Isoleucine and L-Allo-Isoleucine under collision-induced dissociation tandem mass spectrometry. *Analyst* 145, 6632–6638. doi: 10.1039/D0AN00778A

Kong, J., Liu, L., Gao, Y., Chen, S., Li, L., Shu, Y., et al. (2020). Characteristic chemical profile of Juhe Fang extract with lipid-lowering properties. *J. Tradit. Chin. Med. Sci*. 7, 233–244. doi: 10.1016/j.jtcms.2020.07.003

Lv, M., Wang, Y., Wan, X., Han, B., Yu, W., Liang, Q., et al. (2022). Rapid Screening of Proanthocyanidins from the Roots of Ephedra sinica Stapf and its Preventative Effects on Dextran-Sulfate-Sodium-Induced Ulcerative Colitis. *Metabolites* 12, 957. doi: 10.3390/metabo12100957

Liu, X., Wang, X., Yang, Y., Xu, R., Meng, F., Yu, N., et al. (2015). Qualitative and Quantitative Analysis of Lignan Constituents in Caulis Trachelospermi by HPLC-QTOF-MS and HPLC-UV. *Molecules* 20, 8107–8124. doi: 10.3390/molecules20058107

Li, Y., Tan, Y., Wei, N., and Zhang, J. (2016). Diuretic and anti-diuretic bioactivity differences of the seed and shell extracts of *Alpinia oxyphylla* fruit. *Afr. J. Tradit. Complement. Altern. Med.* 13, 25–32. doi: 10.21010/ajtcam.v13i5.4

Lu, A., Jiang, Y., Wu, J., Tan, D., Qin, L., Lu, Y., et al. (2022). Opposite trends of glycosides and alkaloids in Dendrobium nobile of different age based on UPLC-Q/TOF-MS combined with multivariate statistical analyses. *Phytochem. Anal.* 33, 619–634. doi: 10.1002/pca.3115

Ma, Y., Wang, C., Wang, F., Wang, M., Tian, W., Wu, L., et al. (2022). Rapid identification of chemical components of *Folium Photiniae* based on UPLC-Q-TOF-MS. *Chin. Tradit. Herb. Drugs* 53, 6401–6411.

Ma, Y., Tian, T., Xie, W., Jin, Y., Xu, H., Zhang, L., et al. (2016). Major phenolic acids in *Usneae Filum* by UHPLC-Triple-TOF-MS. *Chin. Tradit. Herb. Drugs* 47, 392–400.

Nikolic, D., Li, Y., Chadwick, L., Pauli, G., and van Breemen, R. (2005). Metabolism of xanthohumol and isoxanthohumol, prenylated flavonoids from hops (*Humulus lupulus* L.), by human liver microsomes. *J. Mass Spectrom.* 40, 289–299. doi: 10.1002/jms.753

Ostrowski, W., Wojakowska, A., Grajzer, M., and Stobiecki, M. (2014). Mass spectrometric behavior of phenolic acids standards and their analysis in the plant samples with LC/ESI/MS system. *J. Chromatogr. B* 967, 21–27. doi: 10.1016/j.jchromb.2014.07.005

Pan, H., Shi, F., Fang, C., and Shi, J. (2022). Metabolic characterization of a potent natural neuroprotective agent dendrobine in vitro and in rats. *Acta Pharmacol. Sin*. 43, 1059–1071. doi: 10.1038/s41401-021-00690-9

Rontani, J., Aubert, C., and Belt, S. (2015). EIMS Fragmentation Pathways and MRM Quantification of 7α/β-Hydroxy-Dehydroabietic Acid TMS Derivatives. *J. Am. Soc. Mass Spectrom.* 26, 1606–1616. doi: 10.1007/s13361-015-1157-3

Ren, Z., Nie, B., Liu, T., Yuan, F., Feng, F., Zhang, Y., et al. (2016). Simultaneous Determination of Coumarin and Its Derivatives in Tobacco Products by Liquid Chromatography-Tandem Mass Spectrometry. *Molecules* 21, 1511. doi: 10.3390/molecules21111511

Shahzad, M. N., Ahmad, S., Tousif, M. I., Ahmad, I., Rao, H., Ahmad, B., et al. (2022). Profiling of phytochemicals from aerial parts of *Terminalia neotaliala* using LC-ESI-MS2 and determination of antioxidant and enzyme inhibition activities. *PLOS ONE* 17, e266094. doi: 10.1371/journal.pone.0266094.

Syahbirin, G., Chahyaningtias, A., Radita, R., Ilmiawati, A. (2020). Secondary metabolites of *Temu Putih* (*Curcuma zedoaria*) rhizome. *AIP Conference Proceedings* 2243, 30026. doi: 10.1371/journal.pone.0266094

Scheubert, K., Hufsky, F., and Böcker, S. (2013). Computational mass spectrometry for small molecules. *J. Cheminform.* 5, 12. doi: 10.1371/journal.pone.0266094

Scigelova, M., Hornshaw, M., Giannakopulos, A., and Makarov, A. (2011). Fourier Transform Mass Spectrometry. *Mol. Cell. Proteom.* 10, M111.009431. doi: 10.1074/mcp.M111.009431

Shi, M., Gao, T., Zhang, T., and Han, H. (2019). Characterization of curcumin metabolites in rats by ultra-high-performance liquid chromatography with electrospray ionization quadrupole time-of-flight tandem mass spectrometry. *Rapid Commun. Mass Spectrom.* 33, 1114–1121. doi: 10.1002/rcm.8450

Sinosaki, N., Tonin, A., Ribeiro, M., Poliseli, C., Roberto, S., Silveira, R., et al. (2020). Structural study of phenolic acids by triple quadrupole mass spectrometry with electrospray ionization in negative mode and H/D isotopic exchange. *J. Braz. Chem. Soc.* 31, 402–408. doi: 10.21577/0103-5053.20190197

Tao, J. (2021). *Study on Chemical Substances and Pharmacodynamics of Malt Alkaloids Based on UPLC-Q-TOF-MS/MS and Network Pharmacology* (Wuhan: Hubei University of Traditional Chinese Medicine). doi: 10.27134/d.cnki.ghbzc.2021.000306

Tan, S., Rupasinghe, T., Tull, D., Boughton, B., Oliver, C., McSweeny, C., et al. (2014). Degradation of Curcuminoids by in Vitro Pure Culture Fermentation. *J. Agric. Food Chem*. 62, 11005–11015. doi: 10.1021/jf5031168

Taylor, V. F., March, R. E., Longerich, H. P., and Stadey, C. J. (2005). A mass spectrometric study of glucose, sucrose, and fructose using an inductively coupled plasma and electrospray ionization. *Int. J. Mass Spectrom.* 243, 71–84. doi: 10.1016/j.ijms.2005.01.001

Teso Vilar, E., Fraile, A., Cerero, S., Martínez-Ruiz, P., and Jiménez, F. (2011). Electron ionization mass spectral studies of bridgehead-substituted norbornan-2-ones: camphor derivatives. *Rapid Commun. Mass Spectrom.* 25, 395–409. doi: 10.1002/rcm.4839

Tian, Y., Ma, B., Liu, C., Zhao, X., Yu, S., Li, Y., et al. (2022). Integrated Solid-Phase Extraction, Ultra-High-Performance Liquid Chromatography–Quadrupole-Orbitrap High-Resolution Mass Spectrometry, and Multidimensional Data-Mining Techniques to Unravel the Metabolic Network of Dehydrocostus Lactone in Rats. *Molecules* 27, 7688. doi: 10.3390/molecules27227688

Valgimigli, L., Gabbanini, S., and Matera, R. (2012). “Analysis of maltose and lactose by UHPLC-ESI-MS/MS” in *Dietary Sugars: Chemistry, Analysis, Function and Effects*. Ed. R. P. Victor (Cambridge: Royal Society of Chemistry ), 443–463.

Weinert, C., Wiese, S., Rawel, H., Esatbeyoglu, T., Winterhalter, P., Homann, T., et al. (2012). Methylation of Catechins and Procyanidins by Rat and Human Catechol-O-Methyltransferase: Metabolite Profiling and Molecular Modeling Studies. *Drug Metab. Dispos.* 40, 353–359. doi: 10.1124/dmd.111.041871

Wong-Paz, J., Guyot, S., Aguilar-Zárate, P., Muñiz-Márquez, D., Contreras-Esquivel, J., and Aguilar, C. (2021). Structural characterization of native and oxidized procyanidins (condensed tannins) from coffee pulp (*Coffea arabica*) using phloroglucinolysis and thioglycolysis-HPLC-ESI-MS. *Food Chem.* 340, 127830. doi: 10.1016/j.foodchem.2020.127830

Wang, Y. (2015). *Studies on analysis of flavonoids of flowers, stems and leaves of Abelmoschus manihot (L.) Medicus* (Beijin: Beijing University of Chinese Medicine).

Xu, Y., Liang, P., Chen, X., Gong, M., Zhang, L., Qiu, X., et al. (2021). The Impact of *Citrus*-Tea Cofermentation Process on Chemical Composition and Contents of Pu-Erh Tea: An Integrated Metabolomics Study. *Front. Nutr.* 8, 737539. doi: 10.3389/fnut.2021.737539

Yin, J., Ma, Y., Liang, C., Gao, J., Wang, H., and Zhang, L. (2019). A Systematic Study of the Metabolites of Dietary Acacetin in Vivo and in Vitro Based on UHPLC-Q-TOF-MS/MS Analysis. *J. Agric. Food Chem.* 67, 5530–5543. doi: 10.1021/acs.jafc.9b00330

Yuan, L., Liang, C., Diao, X., Cheng, X., Liao, M., and Zhang, L. (2018). Metabolism studies on hydroxygenkwanin and genkwanin in human liver microsomes by UHPLC-Q-TOF-MS. *Xenobiotica* 48, 332–341. doi: 10.1080/00498254.2017.1319991

Yang, P., Gao, R., Liu, Z., Qu, Q., Yang, C., Shi, X., et al. (2020). Analysis of chemical constituents and six compounds in Qu-feng-sheng-shi Granules via HPLC–ESI–Q/TOF–MSn and HPLC–UV technique. *Biomed. Chromatogr.* 34, e4829. doi:10.1002/bmc.4829

Ying, Y., Yu, M., Xiao, J., and Shen, Q. (2021). Metabolites and Metabolic Pathway Analysis of Germacrone in Rats by UHPLC-Q-Orbitrap HRMS. Chin. *J. Mod. Appl. Pharm.* 38, 430–438. doi: 10.13748/j.cnki.issn1007-7693.2021.04.009

Zheng, G., Liu, M., Chao, Y., Yang, Y., Zhang, D., Tao, Y., et al. (2020). Identification of lipophilic components in Citri Reticulatae Pericarpium cultivars by supercritical CO_2_ fluid extraction with ultra-high-performance liquid chromatography–Q Exactive Orbitrap tandem mass spectrometry. *J. Separat. Sci.* 43, 3421–3440. doi: 0.1002/jssc.202000490

Zhang, K., Shen, Y., Zhang, L., Zhang, Q., Meng, X., Ge, Z., et al. (2022). Rapid establishment of a database of potential quality markers (Q-Marker) of traditional Chinese medicine in *Curcuma Radix* based on UPLC-Triple-TOF-MS and network pharmacology. *Chin. Tradit. Herb. Drugs* 53, 2612–2622.

Zhang, X., Liao, M., Cheng, X., Liang, C., Diao, X., and Zhang, L. (2018). Ultrahigh-performance liquid chromatography coupled with triple quadrupole and time-of-flight mass spectrometry for the screening and identification of the main flavonoids and their metabolites in rats after oral administration of *Cirsium japonicum.* *Rapid Commun. Mass Spectrom.* 32, 1451–1461. doi: 10.1002/rcm.8161

Zhang, Y., Bo, C., Fan, Y., An, R., Chen, L., Zhang, Y., et al. (2019). Qualitative and quantitative determination of *Atractylodes* rhizome using ultra-performance liquid chromatography coupled with linear ion trap-Orbitrap mass spectrometry with data-dependent processing. *Biomed. Chromatogr.* 33, e4443. doi: 10.1002/bmc.4443

Zhang, J., Huang, Z., Qiu, X., Yang, Y., Zhu, D., and Xu, W. (2012). Neutral Fragment Filtering for Rapid Identification of New Diester-Diterpenoid Alkaloids in Roots of *Aconitum carmichaeli* by Ultra-High-Pressure Liquid Chromatography Coupled with Linear Ion Trap-Orbitrap Mass Spectrometry. *PLOS ONE* 7, e52352. doi: 10.1371/journal.pone.0052352
